# Bilocal Field Theory for Composite Scalar Bosons

**DOI:** 10.3390/e26020146

**Published:** 2024-02-08

**Authors:** Christopher T. Hill

**Affiliations:** Particle Theory Department, Fermi National Accelerator Laboratory, P. O. Box 500, Batavia, IL 60510, USA; hill@fnal.gov

**Keywords:** bound states, compositeness, coloron

## Abstract

We give a bilocal field theory description of a composite scalar with an extended binding potential that reduces to the Nambu–Jona-Lasinio (NJL) model in the pointlike limit. This provides a description of the internal dynamics of the bound state and features a static internal wave function, $\phi (\vec{r})$, in the center-of-mass frame that satisfies a Schrödinger–Klein–Gordon equation with eigenvalues $m^{2}$. We analyze the “coloron” model (single perturbative massive gluon exchange) which yields a UV completion of the NJL model. This has a BCS-like enhancement of its interaction, $\propto N_{c}$ the number of colors, and is *classically critical* with $g_{critical}$ remarkably close to the NJL quantum critical coupling. Negative eigenvalues for $m^{2}$ lead to spontaneous symmetry breaking, and the Yukawa coupling of the bound state to constituent fermions is emergent.

## 1. Introduction

Many years ago, Yukawa proposed a multilocal field theory for the description of relativistic bound states [1,2,3]. For a composite scalar field, consisting of a pair of constituents, he introduced a complex bilocal field, Φ(x,y). This is factorized, Φ(x,y)→χ(X)ϕ(r) where $X^{\mu }=\left(x^{\mu }\;+\;y^{\mu }\right)/2$ where $r^{\mu }=\left(x^{\mu }\;-\;y^{\mu }\right)/2$, and χ(X) describes the center-of-mass motion like any conventional point-like field. Then, ϕ(r) describes the internal structure of the bound state. The formalism preserves Lorentz covariance, though we typically “gauge fix” to the center-of-mass frame, and Lorentz covariance is then not manifested. Here, we must confront the issue of “relative time”.

Each of the constituent particles in a relativistic bound state carries its own local clock, e.g., $x^{0}$ and $y^{0}$. These are, in principle, independent; so, the question “how can a description of a multi-particle bound state be given in a quantum theory with a single time variable, $X^{0}$?” arises. To answer this, Yukawa introduced an imaginary “relative time” $r^{0}=\left(x^{0}-y^{0}\right)/2$, but this did not seem to be effective and is an element of his construction we will abandon.

A bilocal field theory formalism can be constructed in an action by considering general properties of free field bilocal actions. However, we can “derive” the bilocal theory from a local constituent field theory by matching the conserved currents of the composite theory with those of the constituent theory. This leads to the removal of relative time, which then becomes associated with canonical normalization of the constituent fields χ and ϕ. In the center-of-mass frame, the internal wave function reduces to a static field, $\phi (\vec{r})$, where $\vec{r}=\left(\vec{x}-\vec{y}\right)/2$. The approach yields a fairly simple solution to the problem of relative time, matching the conclusions one obtains from the elegant Dirac Hamiltonian constraint theory [4,5,6,7,8,9,10]. The resulting $\phi (\vec{r})$ then appears as a straightforward result.

After first considering a bosonic construction, we apply this to a theory of chiral fermions with an extended interaction mediated by a perturbative massive gluon, i.e., the “coloron model” [11,12,13,14,15,16]. This provides a UV completion for the Nambu–Jona-Lasinio (NJL) model [17,18], which is recovered in the point-like limit, $\vec{r}\to 0$. This leads to an effective (mass)^2^ Yukawa potential with coupling *g*. We form bound states with mass $m^{2}$, determined as the eigenvalue of a static Schrödinger–Klein–Gordon (SKG) equation for the internal wave function $\phi (\vec{r})$.

A key result of this analysis leads to a departure from the usual NJL model: the coloron model has a nontrivial *classical critical behavior*, $g>g_{c}$, leading to a bound state with a negative $m^{2}$. The classical interaction is analogous to the Fröhlich Hamiltonian interaction in a superconductor and has a BCS-like enhancement of the coupling by a factor of $N_{c}$ (number of colors) [19,20]. Remarkably, we find the classical $g_{c}$ is numerically close to the NJL critical coupling constant which arises in fermion loops.

The scalar bound state develops an effective Yukawa coupling to its constituent fermions, distinct from *g*, that is emergent in the theory. In the point-like limit this matches the NJL coupling when near criticality. However, in general, this depends in detail upon the internal wave function $\phi (\vec{r})$ and potential. In the point-like limit this is determined by ϕ(0) and we recover the NJL model. However, if we are far from the point-like limit in an extended wave function $\phi (\vec{r})$ might suppress the emergent Yukawa coupling, even though the coloron coupling *g* is large.

The description of a relativistic bound state in the rest frame is similar to the eigenvalue problem of the nonrelativistic Schrödinger equation and some intuition carries over. However, the eigenvalue of the static Schrödinger–Klein–Gordon (SKG) equation is $m^{2}$ rather than energy. Hence, a bound state with positive $m^{2}$ is a resonance that can decay to its constituents and has a Lorentz line-shape in $m^{2}$, and thus has a large distance radiative component to its solution that represents incoming and outgoing open scattering states.

If the eigenvalue for $m^{2}$ is negative or tachyonic; contrary to the non-relativistic case, the bound state represents a chiral vacuum instability. This then requires consideration of a quartic interaction of the composite field, $∼\lambda \Phi^{4}$, which is expected to be generated by the loops in the underlying theory. We treat this phenomenologically in the present paper. In the broken symmetry phase, the composite field Φ(x,y) acquires a vacuum expectation value (VEV), 〈Φ〉=v. In the perturbative quartic coupling limit (λ), in the broken phase, $\phi (\vec{r})$ remains localized and the Nambu–Goldstone modes and Brout–Englert–Higgs (BEH) boson retain the common localized solution for their internal wave functions.

## 2. Constructing a Bilocal Composite Theory

### 2.1. Brief Review of the NJL Model

The Nambu–Jona-Lasinio model (NJL) [17,18] is the simplest field theory of a composite scalar boson, consisting of a pair of chiral fermions. A bound state emerges from an assumed point-like four-fermion interaction and is described by local effective field, Φ(x). The effective field arises as an auxiliary field from the factorization of the four-fermion interaction. In the usual formulation of the NJL model, chiral fermions induce loop effects in a leading large $N_{c}$ limit which, through the renormalization group, leads to interesting dynamic phenomena at low energies. We present a brief review of this.

We assume chiral fermions, each with $N_{c}$ “colors” labeled by (a,b,…). A non-confining, point-like chirally invariant $U{\left(1\right)}_{L}\times U{\left(1\right)}_{R}$ interaction then takes the form:

(1) $$\begin{array}{ccc} & & S_{NJL}=\;\int \;d^{4}x\;(i{\bar{\psi }}_{L}^{a}\left(x\right)\partial \;\;\;/\;\;\;\psi_{aL}\left(x\right)+i{\bar{\psi }}_{R}^{a}\left(x\right)\partial \;\;\;/\;\;\;\psi_{aR}\left(x\right)\\ & & +\frac{g^{2}}{M_{0}^{2}}{\bar{\psi }}_{L}^{a}\left(x\right)\psi_{aR}\left(x\right)\;{\bar{\psi }}_{R}^{b}\left(x\right)\psi_{bL}\left(x\right)) \end{array}$$

This can be readily generalized to a $G_{L}\times G_{R}$ chiral symmetry. We then factorize Equation (1) by introducing the local auxiliary field Φ(x) and write for the interaction:

(2) $$\begin{array}{ccc} & & \;\;\;\;\;\;\;\;\;\;\int \;d^{4}x\left(g{\bar{\psi }}_{L}^{a}\left(x\right)\psi_{aR}\left(x\right)\Phi \left(x\right)+h.c.-M_{0}^{2}\Phi^{†}\left(x\right)\Phi \left(x\right)\right) \end{array}$$

We view Equation (2) as the action defined at the high scale μ∼M. Then, following [21], we integrate out the fermions to obtain the effective action for the composite field Φ at a lower scale μ<<M.

The calculation in the large-$N_{c}$ limit and full renormalization group is discussed in detail in [22,23,24]. The leading $N_{c}$ fermion loop yields the result:

(3) $$\begin{array}{ccc} & & \;\;\;\;\;\;\;\;\;\;\;\;\;L_{M}\to L_{\mu }=g\left[{\bar{\psi }}_{R}\psi_{L}\right]\Phi +h.c+Z\partial_{\mu }\Phi^{†}\partial^{\mu }\Phi \\ & & \;-m^{2}\Phi^{†}\Phi -\frac{\lambda }{2}{\left(\Phi^{†}\Phi \right)}^{2} \end{array}$$

where

(4) $$\begin{array}{ccc} & & \;\;\;\;\;\;\;\;\;\;m^{2}=M_{0}^{2}\;-\;\frac{N_{c}g^{2}}{8\pi^{2}}\left(M_{0}^{2}-\mu^{2}\right)\\ & & \;\;\;\;\;\;\;\;\;\;Z=\frac{N_{c}g^{2}}{8\pi^{2}}\ln\left(M_{0}/\mu \right),\;\;\;\lambda =\frac{N_{c}g^{4}}{4\pi^{2}}\ln\left(M_{0}/\mu \right). \end{array}$$

Here, $M_{0}^{2}$ is the UV loop momentum cut-off, and we include the induced kinetic and quartic interaction terms. The one-loop result can be improved by using the full renormalization group [22,23,24]. Hence, the NJL model is driven by fermion loops, which are ∝ℏ intrinsically quantum effects.

**Figure 1:**
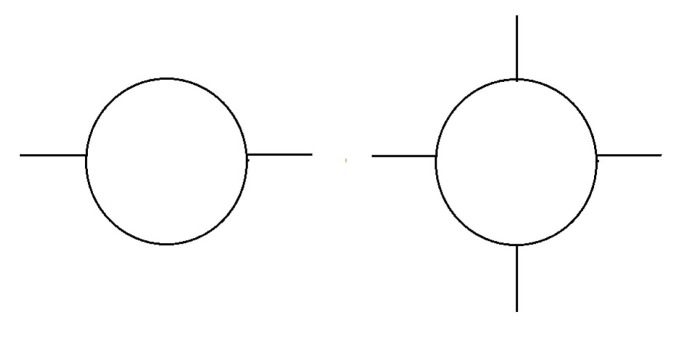
Diagrams contributing to the point-like NJL model effective Lagrangian, Equations (2) and (4). External lines are Φ and internal lines are fermions.

Note the behavior of the composite scalar boson mass, $m^{2}$, of Equation (4) in the UV. The $-N_{c}g^{2}M_{0}^{2}/8\pi^{2}$ term arises from the negative quadratic divergence in the loop involving the pair $\left(\psi_{R},\psi_{L}\right)$ of Figure 1, with UV cut-off $M_{0}^{2}$. Therefore, the NJL model has a critical value of its coupling defined by the cancellation of the large $M_{0}^{2}$ terms for $\mu^{2}=0$

(5) $$\begin{array}{ccc} & & g_{c0}^{2}=\frac{8\pi^{2}}{N_{c}}+O\left(\;\frac{\mu^{2}}{M^{2}}\;\right) \end{array}$$

Note that μ is the running RG mass and comes from the lower limit of the loop integrals and breaks scale invariance and can, in principle, be small. For super-critical coupling, $g>g_{c}^{\prime }$, we see that $m^{2}<0$ and there will be a vacuum instability. The effective action, with a ${\lambda |\Phi |}^{4}$ term, is then the usual sombrero potential. The chiral symmetry is spontaneously broken, the chiral fermions acquire mass, and the theory generates Nambu–Goldstone bosons. Fine-tuning of $g^{2}\approx g_{c}^{2}$ is possible if we want a theory with a hierarchy, $|m^{2}|<\;\;<M_{0}^{2}$.

### 2.2. Construction of Bilocal Compositeness in a Local Scalar Field Theory 

Presently, we obtain a theory of bound states by bilocal fields in a Lorentz invariant model, consisting of a point-like complex scalar field and an interaction mediated by a point-like real field (in Section, we extend this to a chiral fermion interaction via a massive gauge field, analogous to a heavy gluon, aka “coloron”; in Appendix A we give a summary of notation and formulas). Our present treatment will be semi-classical.

Consider local scalar fields φ(x) (complex) and A(x) (real) and action:

(6) $$\begin{array}{ccc} & & \;\;\;\;\;\;\;\;\;\;\;\;\;S=\int_{x}\left({\left|\partial \varphi \right|}^{2}\;+\;\frac{1}{2}{\left(\partial A\right)}^{2}\;-\;\frac{1}{2}M^{2}A^{2}\;-{\;gM|\varphi |}^{2}A\;-\;\frac{\lambda }{2}{\left|\varphi \right|}^{4}\right) \end{array}$$

where we abbreviate ${\left|\partial \varphi \right|}^{2}=\partial_{\mu }\varphi^{†}\partial^{\mu }\varphi$ and ${\left(\partial A\right)}^{2}=\partial_{\mu }A\partial^{\mu }A$. Here, *g* is dimensionless and we refer all mass scales to the single scale *M*. We will discuss the quartic term separately below, and presently set it aside, λ=0.

If we integrate out *A*, we obtain an effective, attractive, bilocal potential interaction term at leading order in $g^{2}$,

(7) $$\begin{array}{ccc} & & \;\;\;\;\;\;\;\;\;\;\;\;\;\;\;\;\;\;\;\;S=\;\;\int_{x}\;{\left|\partial \varphi \right|}^{2}+\frac{g^{2}M^{2}}{2}\;\;\;\int_{xy}\;\;\;\;\varphi^{†}\;\left(y\right)\varphi \left(y\right)D_{F}\left(y\;-\;x\right)\varphi^{†}\;\left(x\right)\phi \left(x\right) \end{array}$$

where the two-point function is given by (i)× the Feynman propagator,

(8) $$\begin{array}{ccc} & & D_{F}\left(x-y\right)=-\;\;\int \frac{e^{iq_{\mu }\left(x^{\mu }-y^{\mu }\right)}}{\left(q^{2}-M^{2}\right)}\frac{d^{4}q}{{\left(2\pi \right)}^{4}} \end{array}$$

The equation of motion of φ is therefore

(9) $$\begin{array}{ccc} & & \;\;\;\;\;\;\;\;\;\;\;\;\;\;\;\;\;\;\;\;\partial^{2}\varphi \left(x\right)-g^{2}M^{2}\;\;\int_{y}\;\;\varphi^{†}\left(y\right)\varphi \left(x\right)D_{F}\left(x-y\right)\varphi \left(y\right)=0 \end{array}$$

(note we have transposed $\varphi^{†}\left(y\right)$ and φ(y) under the integral). In the action in Equation (7), the kinetic term is still local while the interaction is bilocal, and the theory is still classical in that this only involved a tree diagram that is $O(\hbar^{0})$.

We now define a bilocal field of mass dimension d=1

(10) $$\begin{array}{ccc} & & \Phi \left(y,x\right)=M^{-1}\varphi \left(y\right)\varphi \left(x\right). \end{array}$$

The free particle states described by the bilocal field trivially satisfy an equation of motion and a symmetry

(11) $$\begin{array}{ccc} & & \partial_{x}^{2}\Phi \left(x,y\right)=0\;\Phi \left(x,y\right)=\Phi \left(y,x\right) \end{array}$$

and this is generated by a bilocal action

(12) $$\begin{array}{ccc} & & \;\;\;\;\;\;\;\;\;\;S=M^{4}\;\;\int_{xy}\;\;Z{\left|\partial_{x}\Phi \left(x,y\right)\right|}^{2} \end{array}$$

where we will specify the normalization, *Z*, and scale *M* subsequently (we discuss the general properties of the bilocal fields and actions in Appendix B.2 and Appendix B.3). With the bilocal field the interaction of Equation (7), it becomes

(13) $$\begin{array}{ccc} & & \frac{g^{2}M^{4}}{2}\;\;\;\int_{xy}\;\;\;\;\Phi^{†}\left(x,y\right)D_{F}\left(x\;-\;y\right)\Phi \left(x,y\right) \end{array}$$

We can therefore postulate a bilocalized action as a free particle part plus the interaction

(14) $$\begin{array}{ccc} & & \;\;\;\;\;\;\;\;\;\;\;S=\;\;\int_{xy}\;\;\;\left(ZM^{4}{\left|\partial_{x}\Phi \left(x,y\right)\right|}^{2}\;+\;\frac{1}{2}g^{2}M^{4}\Phi^{†}\left(x,y\right)D_{F}\left(x-y\right)\Phi \left(x,y\right)\right) \end{array}$$

In the limit g=0, the field Φ(x,y) and the action faithfully represents two-particle kinematics, and we have the equation of motion

(15) $$\begin{array}{ccc} & & \;\;\;\;\;\;\;\;\;\;0=Z\partial_{x}^{2}\Phi \left(x,y\right)-\frac{1}{2}g^{2}D_{F}\left(x-y\right)\Phi \left(x,y\right) \end{array}$$

We see that a U(1) conserved Noether current is generated by $\Phi \left(x,y\right)\to e^{i\theta (x)}\Phi \left(x,y\right)$

(16) $$\begin{array}{ccc} & & J_{\Phi \mu }\left(x\right)=iZ\int d^{4}y\left(\Phi^{†}\left(x,y\right)\frac{\overset{↔}{\partial }}{\partial x^{\mu }}\Phi \left(x,y\right)\right) \end{array}$$

where $A\overset{↔}{\partial }B=A\partial B-\left(\partial A\right)B$. This must match the conserved U(1) current in the constituent theory

(17) $$\begin{array}{ccc} & & J_{\varphi \mu }\left(x\right)=i\varphi^{†}\left(x\right)\frac{\overset{↔}{\partial }}{\partial x^{\mu }}\varphi \left(x\right) \end{array}$$

Substituting Equation (10) into $J_{\Phi \mu }\left(x\right)$, we see that the matching requires

(18) $$\begin{array}{ccc} & & J_{\Phi \mu }\left(x\right)=J_{\varphi \mu }\left(x\right)\;ZM^{2}\;\;\int \;d^{4}y{\left|\varphi \left(y\right)\right|}^{2} \end{array}$$

Hence

(19) $$\begin{array}{ccc} & & 1=ZM^{2}\int \;d^{4}y\;{\left|\varphi \left(y\right)\right|}^{2} \end{array}$$

This is a required constraint for the bound state sector of the theory. Note that the square of the constraint is the four-normalization of Φ

(20) $$\begin{array}{ccc} & & \;\;\;\;\;\;\;\;\;\;1=Z^{2}M^{4}\int \;d^{4}y\;d^{4}{y\;|\varphi \left(y\right)|}^{2}{\left|\varphi \left(x\right)\right|}^{2}\\ & & =Z^{2}M^{6}\int \;d^{4}y\;d^{4}y\;{\left|\Phi \left(x,y\right)\right|}^{2} \end{array}$$

This implies that the presence of a correlation in the two-particle sector, Φ(x,y), acts as a constraint on the single particle action in that sector. We can now see how the underlying φ action of Equation (7) leads to the Φ action by inserting the constraint of Equation (19) into the kinetic term of Equation (7) and rearranging to obtain

(21) $$\begin{array}{ccc} & & \;\;\;\;\;\;\;\;\;\;S=\;\;\int_{xy}\;\;(ZM^{2}{\left|\varphi \left(y\right)\partial_{x}\varphi \left(x\right)\right|}^{2}\\ & & \;\;\;\;+\frac{g^{2}M^{2}}{2}\varphi^{†}\left(y\right)\varphi \left(x\right)D_{F}\left(x-y\right)\varphi^{†}\left(x\right)\varphi \left(y\right)) \end{array}$$

and *S* remains dimensionless. With the bilocal field of Equation (10) the bilocalized action Equation (21) becomes Equation (12).

Following Yukawa, we go to barycentric coordinates (X,r)

(22) $$\begin{array}{ccc} & & \;\;\;\;\;\;\;\;\;\;X=\frac{1}{2}\left(x+y\right),\;r=\frac{1}{2}\left(x-y\right). \end{array}$$

where $r^{\mu }=\left(r^{0},\vec{r}\right)$, where $\vec{r}$ is the radius and $r^{0}$ is the relative time (Yukawa preferred to write things in terms of ρ=2r, which has the advantage of a unit Jacobian, $\int d^{4}xd^{4}y=$$J\int d^{4}Xd^{4}\rho$ with J=1. We find that the radius, *r*, is more convenient in loop calculations and derivatives are symmetrical, $\partial_{X,r}=\left(\partial_{x}\pm \partial_{y}\right)/2$ vs. $=\frac{1}{2}\partial_{X}+\partial_{\rho }$, but require the Jacobian. See Appendix A for a summary of notation).

Hence, we write

(23) $$\begin{array}{ccc} & & \;\;\;\;\;\;\;\;\;\;\Phi (x,y)=\Phi (X\;+\;r,X\;-\;r)\equiv \Phi (X,r) \end{array}$$

Let $S=S_{K}+S_{P}$ and we can then rewrite the kinetic term, $S_{K}$, using the derivative $\partial_{x}=\frac{1}{2}\left(\partial_{X}\;+\;\partial_{r}\right)$

(24) $$\begin{array}{ccc} & & \;\;\;\;\;\;\;\;\;\;S_{K}=\frac{JM^{4}}{4}\;\;\int_{Xr}\;\;Z{\left|\left(\partial_{X}\;+\;\partial_{r}\right)\Phi \left(X,r\right)\right|}^{2}\;\\ & & \;\;\;\;\;\;\;\;\;\;\;\;\;\;\;=\frac{JM^{4}}{4}\;\;\int_{Xr}\;\left(Z|\partial_{X}{\Phi |}^{2}\;+\;Z{\left|\partial_{r}\Phi \right|}^{2}\;+\;Z\left(\partial_{X}\Phi^{†}\partial_{r}\Phi +h.c.\right)\right) \end{array}$$

Note the Jacobian J=16

(25) $$\begin{array}{ccc} & & J^{-1}=\left|\frac{\partial (X,r)}{\partial (x,y)}\right|={\left(\frac{1}{2}\right)}^{\;\;4}. \end{array}$$

Likewise, the potential term is

(26) $$\begin{array}{ccc} & & S_{P}=\frac{JM^{4}}{2}\;\int_{Xr}\;g^{2}D_{F}\left(2r\right){\left|\Phi \left(X,r\right)\right|}^{2}. \end{array}$$

We will treat the latter term in Equation (24) $Z(\partial_{X}\Phi^{†}\partial_{r}\Phi +h.c.),$ as a constraint, with its contribution to the equation of motion

(27) $$\begin{array}{ccc} & & \frac{\partial }{\partial X^{\mu }}\frac{\partial }{\partial r_{\mu }}\Phi =0 \end{array}$$

We can redefine this term in the action as a Lagrange multiplier while preserving Lorentz invariance

(28) $$\begin{array}{ccc} & & \to \int_{Xr}\eta {\left(\frac{\partial \Phi^{†}}{\partial X^{\mu }}\frac{\partial \Phi }{\partial r_{\mu }}+h.c.\right)}^{2}\;\;\mathrm{hence},\;\;\delta S/\delta \eta =0 \end{array}$$

which also enforces the constraint on a path integral in analogy to gauge fixing. In the following, we assume the constraint is present in the total action but not written explicitly. We therefore have the bilocal action with the constraint understood:

(29) $$\begin{array}{ccc} & & \;\;\;\;\;\;\;\;\;\;\;\;\;\;\;\;\;\;\;\;S=S_{K}+S_{P}=\\ & & \;\;\;\;\;\;\;\;\;\;\;\;\;\;\;\;\;\;\;\;\;\;\;\frac{JM^{4}}{4}\;\;\int_{Xr}\;\;\;(Z|\partial_{X}{\Phi |}^{2}+Z|\partial_{r}{\Phi |}^{2}\;+2g^{2}D_{F}{\left(2r\right)|\Phi \left(X,r\right)|}^{2}) \end{array}$$

Following Yukawa, we factorize Φ (these factorized solutions form a complete set of basis functions)

(30) $$\begin{array}{ccc} & & \sqrt{J/4}\;\Phi \left(X,r\right)=\chi \left(X\right)\phi \left(r\right) \end{array}$$

where ϕ is the internal wave function which we define to be dimensionless, d=0, while χ is an ordinary local field with mass dimension d=1. χ(X) determines the center-of-mass motion of the composite state. The full action for the factorized field takes the form

(31) $$\begin{array}{ccc} & & \;\;\;\;\;\;\;\;\;\;\;\;\;\;\;\;\;\;\;\;S=M^{4}\;\;\int_{Xr}\;(\;Z|\partial_{X}{\chi |}^{2}\left|\phi^{2}\right|\\ & & \;\;\;\;+{\left|\chi \right|}^{2}\left(Z\right|\partial_{r}{\phi |}^{2}\;+2g^{2}D_{F}{\left(2r\right)|\phi \left(r\right)|}^{2}\;)) \end{array}$$

The matching of the U(1) current generated by $\chi \to e^{i\theta (X)}\chi$ (or to have a canonical normalization of χ(X)), where we see that the normalization of the world-scalar four-integral is

(32) $$\begin{array}{ccc} & & 1=ZM^{4}\;\int \;d^{4}r\;{\left|\phi \left(r\right)\right|}^{2} \end{array}$$

where ϕ replaces φ in Equation (19).

We can then represent *S* in terms of two “nested” actions. For the field χ

(33) $$\begin{array}{ccc} & & \;\;\;\;\;\;\;\;\;\;S=\;\int_{X}\;\left(|\partial_{X}{\chi |}^{2}-m^{2}{\left|\chi \right|}^{2}\right)\;\;\;\mathrm{where}\;\;\;m^{2}=-S_{\phi } \end{array}$$

and $S_{\phi }$ is an action for the internal wave function

(34) $$\begin{array}{ccc} & & \;\;\;\;\;\;\;\;\;\;\;\;\;S_{\phi }=M^{4}\;\;\int_{r^{0},\vec{r}}\;\left(\;Z|\partial_{r}\phi \left(r^{\mu }\right){|}^{2}\;+2g^{2}D_{F}\left(2r^{\mu }\right){\left|\phi \left(r^{\mu }\right)\right|}^{2}\;\right) \end{array}$$

Equation (33) then implies

(35) $$\begin{array}{ccc} & & \partial_{X}^{2}\chi =-m^{2}\chi \;\;\;\mathrm{hence},\;\;\chi ∼\exp\left(iP_{\mu }X^{\mu }\right) \end{array}$$

χ(X) has free plane wave solutions with $P^{2}=m^{2}$.

In the center-of-mass frame of the bound state, we can choose χ to have four-momentum $P_{\mu }=\left(m,0,0,0\right)$ where we then have

(36) $$\begin{array}{ccc} & & \Phi \left(X,r\right)=\chi \left(X\right)\phi \left(r^{\mu }\right)\propto \exp\left(imX^{0}\right)\phi \left(r^{\mu }\right). \end{array}$$

ϕ(r) must then satisfy the Lagrange multiplier constraint

(37) $$\begin{array}{ccc} & & P^{\mu }\frac{\partial }{\partial r^{\mu }}\phi \left(r^{\mu }\right)=0 \end{array}$$

and therefore becomes a *static function* of $r^{\mu }=\left(0,\vec{r}\right)$.

While we have specified *Z* in Equation (32), we still have the option of normalizing the internal wave function $\phi (\vec{r})$. This can be conveniently normalized in the center-of-mass frame as

(38) $$\begin{array}{ccc} & & \;\;\;\;\;\;\;\;\;\;\;\;\;\;\;M^{3}\;\int \;\;d^{3}r\;{\left|\phi \left(\vec{r}\right)\right|}^{2}=1 \end{array}$$

Note that in Equation (38), we have implicitly defined the static internal wave function $\phi (\vec{r})$ to be dimensionless, d=0.

We see that the relative time now emerges in the four-integral over ${\left|\phi \left(r\right)\right|}^{2}$ of Equation (32) together with Equation (38)

(39) $$\begin{array}{ccc} & & \;\;\;\;\;\;\;\;\;\;\;\;\;\;\;\;\;\;\;\;1=ZM^{4}\;\;\int \;\;d^{4}{r|\phi \left(r\right)|}^{2}=ZM^{4}\;\;\int \;\;dr^{0}\;\;\;\int \;\;d^{3}r{\left|\phi \left(\vec{r}\right)\right|}^{2}=ZMT \end{array}$$

where $\int dr^{0}=\int dr^{\mu }P_{\mu }/m\equiv T$. Then, from Equation (39), we have

(40) $$\begin{array}{ccc} & & TZ=M^{-1} \end{array}$$

With static $\phi \left(r\right)\to \phi \left(\vec{r}\right)$, the internal action of Equation (34) becomes

(41) $$\begin{array}{ccc} & & \;\;\;\;\;\;\;\;\;\;S_{\phi }=M^{4}\;\;\int_{r^{0},\vec{r}}\;\left(\;-Z|\nabla_{\vec{r}}\phi \left(\vec{r}\right){|}^{2}\;+2g^{2}D_{F}\left(2r^{\mu }\right){\left|\phi \left(\vec{r}\right)\right|}^{2}\;\right) \end{array}$$

where $|\nabla_{\vec{r}}\phi \left(\vec{r}\right){|}^{2}=\nabla_{\vec{r}}\phi^{†}\cdot \nabla_{\vec{r}}\phi$. Note that $\nabla_{\vec{r}}\phi$ is spacelike, and the arguments of the constrained $\phi (\vec{r})$ are now three-vector; however, $D_{F}\left(2r^{\mu }\right)$ still depends upon the four-vector $r^{\mu }$.

There remains the integral over relative time $r^{0}$ in the action. For the potential, we have the residues

(42) $$\begin{array}{ccc} & & \;\;\;\;\;\;\;\;\;\;\;\;\;\;\;\;\;-V\left(r\right)=2\;\;\int \;\;dr^{0}D_{F}\left(2r\right)=\;\int \;\;\frac{e^{2i\vec{q}\cdot \vec{r}}}{{\vec{q}}^{2}+M^{2}}\frac{d^{3}q}{{\left(2\pi \right)}^{3}}=\;\frac{e^{-2M|\vec{r}|}}{8\pi |\vec{r}|} \end{array}$$

and the potential term in the action becomes the static Yukawa potential

(43) $$\begin{array}{ccc} & & \;\;\;\;\;\;\;\;\;\;S_{P}=-M^{3}\;\;\int_{\vec{r}}\;g^{2}MV\left(\vec{r}\right){\left|\phi \left(\vec{r}\right)\right|}^{2},\;V\left(\vec{r}\right)=-\frac{e^{-2M|\vec{r}|}}{8\pi |\vec{r}|} \end{array}$$

The $\phi (\vec{r})$ kinetic term in Equation (41) becomes

(44) $$\begin{array}{ccc} & & \;\;\;\;\;\;\;\;\;\;S_{K}=-M^{4}\;\;\int_{r^{0},\vec{r}}\;\;Z|\nabla_{\vec{r}}\phi \left(\vec{r}\right){|}^{2}\;=-M^{4}ZT\;\;\int_{\vec{r}}\;{\left|\nabla_{\vec{r}}\phi \left(\vec{r}\right)\right|}^{2}\;\\ & & =-M^{3}\;\;\int_{\vec{r}}\;{\left|\nabla_{\vec{r}}\phi \left(\vec{r}\right)\right|}^{2}\; \end{array}$$

where we use Equation (40). The action $S_{\phi }$ thus becomes

(45) $$\begin{array}{ccc} & & \;\;\;\;\;\;\;\;\;\;\;\;\;m^{2}=-S_{\phi }=M^{3}\int_{\vec{r}}\left(|\nabla_{\vec{r}}{\phi |}^{2}+g^{2}MV\left(r\right){\left|\phi \left(\vec{r}\right)\right|}^{2}\right) \end{array}$$

Note that $S_{\phi }$ has dimension d=2, as it must for $m^{2}$. We thus see, as previously mentioned, that the combination ZT occurs in the theory, and the relative time has disappeared into normalization constraints; see Equations (32) and (38).

The radicalization of $S_{\phi }$ leads to the Schrödinger–Klein–Gordon (SKG) equation in the center-of-mass frame

(46) $$\begin{array}{ccc} & & \;\;\;\;\;\;\;\;\;\;-\nabla_{r}^{2}\phi \left(r\right)-g^{2}M\frac{e^{-2M|\vec{r}|}}{8\pi |\vec{r}|}\phi \left(r\right)=m^{2}\phi \left(r\right). \end{array}$$

where, for spherical symmetry in a ground state,

(47) $$\begin{array}{ccc} & & \nabla_{r}^{2}=\partial_{r}^{2}+\frac{2}{r}\partial_{r} \end{array}$$

We see that the induced mass^2^ of the bound state, $m^{2}$, is the eigenvalue of the SKG equation. We can compare this to a non-relativistic Schrödinger equation (NRSE)

(48) $$\begin{array}{ccc} & & \;\;\;\;\;\;\;\;\;\;-\frac{1}{2M}\nabla_{r}^{2}\phi \left(x\right)-g^{2}\frac{e^{-2Mr}}{16\pi r}\phi =E\phi \left(\vec{r}\right) \end{array}$$

In the next section, we will obtain similar results for a bound state of chiral fermions and use the known results for the Yukawa potential in the NRSE to obtain the critical coupling. The negative eigenvalue of *E* in the NRSE, which signals binding, presently implies a vacuum instability.

Integrating both parts, we then have, from Equation (45)

(49) $$\begin{array}{ccc} & & \;\;\;\;\;\;\;\;\;\;\;\;\;m^{2}=M^{3}\int_{\vec{r}}\left(\phi^{†}\left(-\nabla_{r}^{2}\phi +g^{2}MV\left(r\right)\phi \left(\vec{r}\right)\right)\right) \end{array}$$

Note the consistency, using Equation (9), and the normalization of the dimensionless field ϕ of Equation (38).

More generally, by promoting χ to a (1+3) time-dependent field while maintaining a static ϕ, we have the full joint action:

(50) $$\begin{array}{ccc} & & \;\;\;\;\;\;\;\;\;\;\;S=\;M^{3}\;\;\int_{X\vec{r}}\;\left({\;|\phi |}^{2}\;{\left|\frac{\partial \chi }{\partial X}\right|}^{2}\;\;\;-\;{\left|\chi \right|}^{2}\left(|\nabla_{r}{\phi |}^{2}\;+\;g^{2}MV\left(r\right){\left|\phi \left(r\right)\right|}^{2}\right)\right) \end{array}$$

In summary, we have constructed, by “bilocalization” of a local field theory, a bilocal field description Φ(x,y) for the dynamics of binding a pair of particles. The dynamics implies that, in barycentric coordinates, $\Phi \left(x,y\right)∼\Phi \left(X,r\right)∼\chi \left(X\right)\phi \left(\vec{r}\right)$, where the internal wave function, $\phi (\vec{r})$, is a static function of $\vec{r}$ and satisfies an SKG equation with eigenvalue $m^{2}$, which determines the squared-mass of a bound state. This illustrates the removal of relative time in an action formalism, which is usually framed in the context of Dirac Hamiltonian constraints [4,5].

### 2.3. Simplified Normalization

The normalization system we have thus far used is awkward. We can facilitate this by defining a new integral over the internal wave function three-space $\vec{r}$:

(51) $$\begin{array}{ccc} & & \int_{r}^{\prime }\equiv \int \frac{d^{3}r}{V}\;\;\mathrm{where}\;\;V=M^{-3} \end{array}$$

We then have the key elements of the theory in this notation:

(52) $$\begin{array}{ccc} & & S=\;\int \;\;d^{4}X\left(|\partial_{X}{\chi |}^{2}-m^{2}{\left|\chi \right|}^{2}\right)\\ & & 1=\int_{r}^{\prime }|\phi \left(\vec{r}\right){|}^{2}=\int \;\frac{d^{3}r}{V}{\left|\phi \left(\vec{r}\right)\right|}^{2}\\ & & m^{2}=-S_{\phi }\\ & & S_{\phi }=\int_{r}^{\prime }\left(-|\partial_{\vec{r}}{\phi |}^{2}-g^{2}MV\left(r\right){\left|\phi \left(\vec{r}\right)\right|}^{2}\right)\\ & & V\left(r\right)=-\frac{e^{-2M|\vec{r}|}}{8\pi |\vec{r}|} \end{array}$$

Our general notation is summarized in Appendix A.

## 3. The Coloron Model 

### 3.1. Boundstate and $N_{c}$-Enhanced Coupling 

The point-like NJL model can be viewed as the limit of a physical theory with a bilocal interaction. An example that motivates the origin of the NJL interaction is an analogue of QCD, with a massive and perturbatively coupled gluon. We call this a “coloron model”, and it has been extensively deployed to describe chiral constituent and heavy–light quark models [13,25,26], the possibility of the BEH boson composed of top quarks, and as a generic model for experimental search strategies [11,12,14,15,16,27].

Consider a nonconfining $SU(N_{c})$ gauge Theory with a broken global $SU(N_{c})$, where the coloron gauge fields $A_{\mu }^{A}$ acquire mass *M* and have a fixed coupling constant *g*. We assume chiral fermions, each with $N_{c}$ “colors” labeled by (a,b,…) with the local Dirac action

(53) $$\begin{array}{ccc} & & \;\;\;\;\;\;\;\;\;\;S_{F}=\int_{x}\left(i{\bar{\psi }}_{L}^{a}\left(x\right)D\;\;\;/\;\;\;\psi_{aL}\left(x\right)+i{\bar{\psi }}_{R}^{a}\left(x\right)D\;\;\;/\;\;\;\psi_{aR}\left(x\right)\right) \end{array}$$

where the covariant derivative is

(54) $$\begin{array}{ccc} & & D_{\mu }=\partial_{\mu }-igA_{\mu }^{A}\left(x\right)T^{A} \end{array}$$

and $T^{A}$ are the adjoint representation generators of $SU(N_{c})$. We assume the colorons have a common mass *M*.

The single coloron exchange interaction then takes a bilocal current-current form:

(55) $$\begin{array}{ccc} & & S_{C}=-g^{2}\int_{xy}{\bar{\psi }}_{L}\left(x\right)\gamma_{\mu }T^{A}\psi_{L}\left(x\right)D^{\mu \nu }\left(x-y\right){\bar{\psi }}_{R}\left(y\right)\gamma_{\nu }T^{A}\psi_{R}\left(y\right) \end{array}$$

where $T^{A}$ are generators of $SU(N_{c})$. The coloron propagator in a Feynman gauge yields:

(56) $$\begin{array}{c} D_{\mu \nu }\left(x-y\right)=\int \frac{-ig_{\mu \nu }}{q^{2}-M^{2}}e^{iq(x-y)}\frac{d^{4}q}{{\left(2\pi \right)}^{4}} \end{array}$$

A Fierz rearrangement of the interaction to leading order in $1/N_{c}$ leads to an attractive potential [11,12]:

(57) $$\begin{array}{ccc} & & \;\;\;\;\;\;\;\;\;\;S_{C}=g^{2}\;\;\int_{xy}\;\;\;{\bar{\psi }}_{L}^{a}\left(x\right)\psi_{aR}\left(y\right)\;D_{F}\left(x-y\right)\;{\bar{\psi }}_{R}^{b}\left(y\right)\psi_{bL}\left(x\right) \end{array}$$

where $D_{F}$ is defined in Equation (8). Note that if we suppress the $q^{2}$ term in the denominator of Equation (56)

(58) $$\begin{array}{ccc} & & D_{F}\left(x-y\right)\to \frac{1}{M^{2}}\delta^{4}\left(x-y\right) \end{array}$$

and we immediately recover the point-like NJL model interaction.

Consider spin-0 fermion pairs of a given color $\left[\bar{a}b\right]$ ${\bar{\psi }}_{R}^{a}\left(x\right)\psi_{bL}\left(y\right)\;$. We will have free fermionic scattering states, $:\;{\bar{\psi }}_{R}^{a}\left(x\right)\psi_{bL}\left(y\right)\;:$ coexisting in the action with bound states ∼Φ(x,y)

(59) $$\begin{array}{ccc} & & \;\;\;\;\;\;\;\;\;\;\;\;\;{\bar{\psi }}_{R}^{a}\left(x\right)\psi_{bL}\left(y\right)\;\to M^{2}\;:\;{\bar{\psi }}_{R}^{a}\left(x\right)\psi_{bL}\left(y\right)\;:\;+\;M^{2}\Phi_{\;b}^{a}\left(x,y\right),\;\; \end{array}$$

The normal ordering :…: signifies that we have subtracted the bound state from the product. These will be eigenstates of the equation of motion and will be orthogonal wave functions.

We see that $\Phi_{b}^{a}\left(X,r\right)$ is an $N_{c}\times N_{c}$ complex matrix that transforms as a product of $SU(N_{c})$ representations, ${\bar{N}}_{c}\times N_{c}$, and therefore decomposes into a singlet plus an adjoint representation of $SU(N_{c})$. We write $\Phi_{b}^{a}$ it as a matrix $\tilde{\Phi }$ by introducing the $N_{c}^{2}-1$ adjoint matrices, $T^{A}$, where $\mathrm{Tr}\left(T^{A}T^{B}\right)=\frac{1}{2}\delta^{AB}$. The unit matrix is $T^{0}\equiv \;$diag$\left(1,1,1,\ldots \right)/\sqrt{2N_{c}}$, and $\mathrm{Tr}{\left(T^{0}\right)}^{2}=1/2$, hence we have

(60) $$\begin{array}{ccc} & & \tilde{\Phi }=\sqrt{2}\left(T^{0}\Phi^{0}+\sum_{A}T^{A}\Phi^{A}\right) \end{array}$$

The $\sqrt{2}$ is present because $\Phi^{0}$ and $\Phi^{A}$ form complex representations since they also represent the $U{\left(1\right)}_{L}\times U{\left(1\right)}_{R}$ chiral symmetry.

For the bilocal fields, we have a bosonic kinetic term with the constraint

(61) $$\begin{array}{ccc} & & \;\;\;\;\;\;\;\;\;\;S_{K}=\frac{JZM^{4}}{2}\;\int_{Xr}\;\;\mathrm{Tr}\left(|\partial_{X}\tilde{\Phi }{|}^{2}+|\partial_{r}\tilde{\Phi }{|}^{2}\;+\;\eta {\left|\partial_{X}{\tilde{\Phi }}^{†}\partial_{r}\tilde{\Phi }\right|}^{2}\right) \end{array}$$

Note the numerical factor differs from the scalar case by treating (x,y) symmetrically as in Equation (A15). For the singlet representations this takes the form

(62) $$\begin{array}{ccc} & & \;\;\;\;\;\;\;\;\;\;\;\;\;\;\;\;\;\;\;\;S_{K}=\frac{JZM^{4}}{2}\;\;\int_{Xr}\;\left(|\partial_{X}\Phi^{0}{|}^{2}+|\partial_{r}\Phi^{0}{|}^{2}\;+\;\eta {\left|\partial_{X}\Phi^{0†}\partial_{r}\Phi^{0}\right|}^{2}\right) \end{array}$$

We assume the constraint in the barycentric frame, and integrate out relative time with ZMT=1.

(63) $$\begin{array}{ccc} & & \;\;\;\;\;\;\;\;\;\;\;\;\;\;\;\;\;\;\;\;S_{K}=\left(J/2\right)\;\;\int_{X}\int_{\vec{r}}^{\prime }\;\left(|\partial_{X}\Phi^{0}\left(X,\vec{r}\right){|}^{2}-{\left|\partial_{\vec{r}}\Phi^{0}\left(X,\vec{r}\right)\right|}^{2}\right) \end{array}$$

(where $\int_{\vec{r}}^{\prime }=M^{3}\int d^{3}r$). Factorizing $\Phi^{0}$

(64) $$\begin{array}{ccc} & & \sqrt{J/2}\;\Phi^{0}\left(X,r\right)=\chi \left(X\right)\phi \left(r\right) \end{array}$$

then the kinetic term action becomes identical to the bosonic case

(65) $$\begin{array}{ccc} & & \;\;\;\;\;\;\;\;\;\;\;\;\;\;\;\;\;\;\;\;S_{K}=\;\;\int_{X}\left(|\partial_{X}{\chi \left(X\right)|}^{2}-{\left|\chi \left(X\right)\right|}^{2}\int_{\vec{r}}^{\prime }\;{\left|\partial_{\vec{r}}\phi \left(\vec{r}\right)\right|}^{2}\right) \end{array}$$

with

(66) $$\begin{array}{ccc} & & \int_{\vec{r}}^{\prime }{\left|\phi \left(\vec{r}\right)\right|}^{2}=1 \end{array}$$

If we include the free fermion scattering states, the full bound state interaction of Equation (57) becomes

(67) $$\begin{array}{ccc} & & \;\;\;\;\;\;\;\;\;\;S_{C}\to g^{2}\;\;\int_{xy}\;\;:\;{\bar{\psi }}_{L}^{a}\left(x\right)\psi_{aR}\left(y\right)\;:D_{F}\left(x-y\right):\;{\bar{\psi }}_{R}^{b}\left(y\right)\psi_{bL}\left(x\right)\;:\\ & & +g^{2}JM^{2}\sqrt{N_{c}}\;\;\int_{X,r}\;\;\;:\;{\bar{\psi }}_{L}^{a}\left(X\;-\;r\right)\psi_{aR}\left(X\;+\;r\right)\;:D_{F}\left(2r\right)\;\Phi^{0}+h.c.\\ & & +g^{2}JM^{4}N_{c}\;\;\int_{X,r}\;\;\;\;\;\Phi^{0†}\left(X,r\right)\;D_{F}\left(2r\right)\;\Phi^{0}\left(X,r\right) \end{array}$$

where

(68) $$\begin{array}{ccc} & & D_{F}\left(2r\right)=-\;\int \frac{1}{\left(q^{2}-M^{2}\right)}e^{2iq_{\mu }r^{\mu }}\frac{d^{4}q}{{\left(2\pi \right)}^{4}} \end{array}$$

The leading term $S_{C}$ of Equation (69) is just a free four-fermion scattering state interaction and has the structure of an NJL interaction in the limit of Equation (55). This identifies $g^{2}$ as the NJL coupling constant. This is best treated separately by the local interaction of Equation (57). We therefore omit this term in the discussion of the bound states.

The second term$∼\mathrm{Tr}\left(\psi^{†}\psi \right)\Phi^{0}+h.c.$ in Equation (69) determines the Yukawa interaction between the bound state $\Phi^{0}$ and the free fermion scattering states. We will treat this below.

Note that the third term is the binding interaction and it involves only the singlet, $\mathrm{Tr}\tilde{\Phi }=\sqrt{N_{c}}\Phi^{0}$. It can then be written in Equation (42) as

(69) $$\begin{array}{ccc} & & \;\;\;\;\;\;\;\;\;\;S_{C}\to g^{2}JM^{4}N_{c}\;\;\int_{X,r}\;\;\;\;\;\Phi^{0†}\left(X,r\right)\;D_{F}\left(2r\right)\;\Phi^{0}\left(X,r\right)\\ & & =g^{2}N_{c}\;\int_{X}{\left|\chi \left(X\right)\right|}^{2}\int_{\vec{r}}^{\prime }{\left|\phi \left(\vec{r}\right)\right|}^{2}M\frac{e^{-2M|\vec{r}|}}{8\pi |\vec{r}|} \end{array}$$

We see that adjoint representation $\Phi^{A}$ is decoupled from the interaction and remain as two-body massless scattering states. Hence, they do not form bound states by the interaction.

We also see that the singlet $\Phi^{0}$ singlet field has an enhanced interaction by a factor of $N_{c}$. This is analogous to a BCS superconductor, where the $N_{c}$ color pairs are analogues of *N* Cooper pairs and the weak four-fermion Fröhlich Hamiltonian interaction is enhanced by a factor of $N_{Cooper}$ [19,20]. The color enhancement also occurs in the NJL model, but at loop level. Here, we see that the color enhancement is occurring in the semi-classical (no loop) coloron theory by this coherent mechanism.

Hence, the removal of relative time is then the identical procedure as in the previous model (and absorbs away *Z* and *T* as in Equations (32), (39) and (40)), and leads to the same action, $S=S_{K}+S_{C}$, in the compact notation of Equation (52) with the interaction enhanced by $N_{c}$.

The radicalization of ϕ then leads to the SKG equation

(70) $$\begin{array}{ccc} & & \;\;\;\;\;\;\;\;\;\;-\nabla_{r}^{2}\phi \left(\vec{r}\right)-g^{2}N_{c}M\frac{e^{-2M|\vec{r}|}}{8\pi |\vec{r}|}\phi \left(\vec{r}\right)=m^{2}\phi \left(\vec{r}\right). \end{array}$$

### 3.2. Classical Criticality of the Coloron Model

The coloron model furnishes a direct UV completion of the NJL model. However, in the coloron model, we do not need to invoke large-$N_{c}$ quantum loops to have a critical theory. Rather, it leads to an SKG potential of the Yukawa form which has a *classical critical coupling*, $g_{c}$. For $g<g_{c}$, the theory is subcritical and produces resonant bound states that decay into chiral fermions. For $g>g_{c}$, the theory produces a tachyonic bound state which implies a chiral instability and Φ must develop a VEV. This requires stabilization by, e.g., quartic interactions and a sombrero potential. All of this is treated bosonically in our present formalism.

The criticality of the Yukawa potential in the nonrelativistic Schrödinger equation is discussed in the literature in the context of “screening”. The nonrelativistic Schrödinger equation $r=|\vec{r}|$ is:

(71) $$\begin{array}{ccc} & & -\nabla^{2}\psi -2m\alpha \frac{e^{-\mu r}}{r}\psi =2mE \end{array}$$

and criticality (eigenvalue E=0) occurs for $\mu =\mu_{c}$ where a numerical analysis yields [28,29]

(72) $$\begin{array}{ccc} & & \mu_{c}=1.19\alpha m \end{array}$$

For us, the spherical SKG equation is now $r=|\vec{r}|$

(73) $$\begin{array}{ccc} & & \;\;\;\;\;\;\;\;\;\;-\nabla_{r}^{2}\phi \left(r\right)-g^{2}N_{c}M\frac{e^{-2Mr}}{8\pi r}\phi \left(r\right)=0 \end{array}$$

Comparing, gives us a critical value of the coupling constant, when $\mu_{c}\to 2M$, m→M/2 and $\alpha \to g^{2}N_{c}/8\pi$, then:

(74) $$\begin{array}{ccc} & & \;\;\;\;\;\;\;\;\;\;\;\;\;2M=\left(1.19\right)\left(\;\frac{M}{2}\;\right)\left(\;\frac{g^{2}N_{c}}{8\pi }\;\right),\;\;\mathrm{hence}:\;\;g^{2}/4\pi =6.72/N_{c} \end{array}$$

We can compare the NJL critical value of Equation (4)

(75) $$\begin{array}{ccc} & & g_{cNJL}^{2}/4\pi =2\pi /N_{c}=6.28/N_{c}. \end{array}$$

Hence, the NJL quantum criticality is a comparable effect, with a remarkably similar numerical value for the critical coupling.

Note that we can rewrite Equation (73) with dimensionless coordinates, $\vec{u}=M\vec{r}$, $u=M|\vec{r}|$

(76) $$\begin{array}{ccc} & & M^{2}\left(-\nabla_{u}^{2}\phi \left(u\right)-g^{2}N_{c}\frac{e^{-2u}}{8\pi u}\phi \left(u\right)\right)=0 \end{array}$$

and then $M^{2}$ only appears as an overall scale factor. Hence, we see that critical coupling is determined by Equation (76), and the scale *M* cancels out at criticality. The mass scale *M* is dictated in the exponential $e^{-2Mr}$ of the particular Yukawa potential, together with canonical normalization of ϕ. In general, we can start with the dimensionless coordinate form of the SKG equation and infer the scale *M* by matching it to the potential. In this way, solutions may exist where the scale in the potential is driven by the renormalization group. This will be investigated elsewhere.

However, it is important to realize that the NJL model involves Yukawa coupling, $g_{NJL}$, while the present criticality involves the coloron coupling constant. The NJL coupling is emergent in the coloron model, and we need to compute it.

### 3.3. Yukawa Interaction

The second term in Equation (67) is the induced Yukawa interaction $S_{Y}$, and can be written with the factorized field as:

(77) $$\begin{array}{ccc} & & \;\;\;\;\;\;\;\;\;\;S_{Y}=g^{2}\left(\sqrt{2JN_{c}}\right)M^{2}\times \\ & & \;\;\;\;\;\;\;\;\;\;\int_{Xr}\;:\;{\bar{\psi }}_{L}^{a}\left(X-r\right)\psi_{aR}\left(X+r\right)\;:D_{F}\left(2r\right)\chi \left(X\right)\phi \left(\vec{r}\right)+h.c. \end{array}$$

This is the effective Yukawa interaction between the bound state $\Phi^{0}$ and the free fermion scattering states.

We cannot simply integrate the relative time here. However, we can first connect this to the point-like limit by suppressing the $q^{2}$ term in the denominator of D(2r) with z→0 in:

(78) $$\begin{array}{ccc} & & \;\;\;\;\;\;\;\;\;\;D_{F}\left(2r\right)\to \;\;\int \;\;\frac{1}{M^{2}}e^{2iq_{\mu }r^{\mu }}\frac{d^{4}q}{{\left(2\pi \right)}^{4}}\to \frac{1}{JM^{2}}\delta^{4}\left(r\right) \end{array}$$

where $\delta^{4}\left(2r\right)=J^{-1}\delta^{4}\left(r\right)$, hence with $J^{-1}=1/16$

(79) $$\begin{array}{ccc} & & \;\;\;\;\;\;\;\;\;\;S_{Y}=g^{2}\left(\sqrt{N_{c}/8}\right)\int_{X}\;{\bar{\psi }}_{L}^{a}\left(X\right)\psi_{aR}\left(X\right)\chi \left(X\right)\phi \left(0\right)+h.c. \end{array}$$

This gives a value of the Yukawa coupling

(80) $$\begin{array}{ccc} & & g_{Y}=g^{2}\left(\sqrt{N_{c}/8}\right)\phi \left(0\right) \end{array}$$

The wave function at the origin, ϕ(0), in the NJL limit is somewhat undefined. However, if we consider a spherical cavity of radius *R*, where MR=π/2, with a *confined* and dimensionless ϕ(r), then ϕ(0) is obtained (see [30] Equation (B58))

(81) $$\begin{array}{ccc} & & \phi \left(0\right)=\frac{1}{\pi }. \end{array}$$

Plugging this into the expression for $g_{Y}$ in Equation (80) gives

(82) $$\begin{array}{ccc} & & g_{Y}=g^{2}\sqrt{N_{c}/8\pi^{2}}=g^{2}/g_{cNJL} \end{array}$$

where $g_{cNJL}^{2}=\left(8\pi^{2}/N_{c}\right)$ is the critical coupling of the NJL model, as seen in Equation (4).

Hence, if the coloron coupling constant, $g^{2}$, is critical, as in Equations (74) and (75), we have seen that $g^{2}\approx g_{cNJL}^{2}$, and the induced Yukawa coupling from Equation (82) is then $g_{Y}\approx g_{cNJL}$. The coloron model is then consistent with the NJL model in the point-like limit where the NJL model coupling is the Yukawa coupling, as seen in Equation (2).

However, the induced Yukawa coupling in the bound state, $g_{Y}$, may be significantly different than the coloron coupling *g* in realistic extended $\vec{r}$ models. The result we just obtained applies when we assume the strict point-like limit of $D_{F}\left(2r\right)∼\delta^{3}\left(r\right)$, while in reality, as the potential becomes more extended, the $\int V\left(\vec{r}\right)\phi \left(\vec{r}\right)$ may become smaller, even if the coloron coupling *g* may be supercritical. We anticipate this could have implications for a composite Higgs model, which will be investigated elsewhere (together with loop effects including the extended wave function). There may also be additional new effects that occur at the loop level in extended potentials, such as the infall of zero modes, as suggested in [31].

### 3.4. Spontaneous Symmetry Breaking

For subcritical Coupling, there are resonance solutions with positive $m^{2}$ that have large distance tails of external incoming and outgoing radiation, representing a steady state of resonant production and decay. The portion of the wave function localized within the potential can be viewed as the resonant bound state for normalization purposes, while the large distance tail is non-normalizable radiation.

With super-critical coupling, $g>g_{c}$, the bilocal field Φ(X,r) has a negative squared mass eigenvalue (tachyonic), with a well-defined localized wave function. In the region external to the potential (forbidden zone), the field is exponentially damped. At exact criticality with $g=g_{c}-\epsilon$, there is a 1/r (quasi-radiative) tail that switches to exponential damping for $g=g_{c}+\epsilon$. The supercritical solutions are localized and normalizable over the entire space $\vec{r}$, but with $m^{2}<0$, they lead to exponential runaway in time of the field $\chi (X^{0})$, and must be stabilized, typically with a ${\left|\Phi \right|}^{4}$ interaction.

We then treat the supercritical case as resulting in spontaneous symmetry breaking. In the point-like limit, Φ(X)∼Φ(X,0), the theory has the “sombrero potential”,

(83) $$\begin{array}{ccc} & & V\left(\Phi \right)=-|M^{2}\Phi^{2}|+\frac{\lambda }{2}{\left|\Phi \right|}^{4} \end{array}$$

The point-like field develops a VEV, $\langle \Phi \rangle =\left|M\right|/\sqrt{\lambda }$. In this way, the bound state theory will drive the usual chiral symmetry breaking from the underlying dynamics of a potential induced by new physics.

A quartic potential generally exists in a local field theory, Equation (6), and would be induced by free loops in the coloron model. We can introduce a bilocalized quartic interaction as a model by presently introducing another world scalar factor with coefficient $Z^{\prime }$

(84) $$\begin{array}{ccc} & & \;\;\;\;\;\;\;\;\;\;\frac{\lambda_{0}}{2}\int d^{4}{x|\varphi |}^{4}\to \frac{\lambda_{0}}{2}\int d^{4}y\;Z^{\prime }{\left|\varphi \left(y\right)\right|}^{4}\;\;\int d^{4}x{\left|\varphi \right|}^{4}\\ & & \;\;\;\;\;\;\;\;\;\;=\frac{Z^{\prime }MT\hat{\lambda }}{2}\;\int_{X}\;\;\int_{r}^{\prime }\;\;{\left|\chi \left(X\right)\phi \left(\vec{r}\right)\right|}^{4} \end{array}$$

and $Z^{\prime }MT=1$ to absorb relative time.

The simplest sombrero potential can therefore be modeled as

(85) $$\begin{array}{ccc} & & \;\;\;\;\;\;\;\;\;\;\;S=\;\;\int_{X\vec{r}}^{\prime }{\;(\;|\phi |}^{2}\;{\left|\frac{\partial \chi }{\partial X}\right|}^{2}\;\;\;-{\;|\chi |}^{2}\left(\right|\nabla_{r}{\phi |}^{2}\;+\;g^{2}N_{c}{MV\left(r\right)|\phi \left(r\right)|}^{2})\\ & & \;\;-{\left|\chi \right|}^{4}\frac{\hat{\lambda }}{2}{\left|\phi \left(\vec{r}\right)\right|}^{4}) \end{array}$$

In the case of a perturbatively small λ, we expect the eigensolution of ϕ to be essentially unaffected

(86) $$\begin{array}{ccc} & & \int_{r}^{\prime }\;\;\left(\;|\nabla_{\vec{r}}{\phi |}^{2}\;+\;g^{2}N_{c}\;MV\left(r\right){\left|\phi \left(\vec{r}\right)\right|}^{2}\;\right)\approx m^{2} \end{array}$$

The effective quartic coupling is then further renormalized by the internal wave function

(87) $$\begin{array}{ccc} & & \frac{\hat{\lambda }}{2}{\left|\chi \right|}^{4}\int_{r}^{\prime }|\phi \left(\vec{r}\right){|}^{4}={\left|\chi \right|}^{4}\frac{\tilde{\lambda }}{2} \end{array}$$

In this case, we see that χ develops a VEV in the usual way:

(88) $$\begin{array}{ccc} & & {\langle |\chi |}^{2}\rangle =|m^{2}|/\tilde{\lambda }=v^{2} \end{array}$$

This is a consequence of $\phi (\vec{r})$ remaining localized in its potential.

The external scattering state fermions, $\psi^{a}\left(X\right)$, will then acquire mass through the emergent Yukawa interaction described in the previous section, $∼g_{Y}\langle |\chi |\rangle .$ However, an issue we have yet to resolve is whether the induced fermion masses back-react with the VEV solution itself. We segregated the free fermions from the bound state wave function, Φ, by shifting, so we are presently arguing that Φ forms a VEV as described above, and the scattering state fermions independently acquire mass as spectators, but this may require a more detailed analysis, and for general large $\tilde{\lambda }$ (as in a nonlinear sigma model), the situation is potentially more complicated.

## 4. Summary and Conclusions

In the present paper, we have given a formulation of bilocal field theory, Φ(x,y), as a variation on Yukawa’s original multilocal field theory of composite particles [1,2,3]. In particular, we focus on two-particle-bound states consisting of bosons or chiral fermions and scattering states. There are many foreseeable extensions of the present work. Here, we construct bilocal field theories from an underlying local interacting field theory via the introduction of “world-scalars”. We then go to barycentric coordinates, and the bilocal field is “factorized”

(89) $$\begin{array}{ccc} & & X=\frac{1}{2}\left(x+y\right),\;r=\frac{1}{2}\left(x-y\right)\\ & & \Phi (x,y)\to \Phi (X,r)=\chi (X)\phi (r) \end{array}$$

Here, χ(X) describes center-of-mass motion like any pointlike scalar field, while ϕ(r) is the internal wave function of the bound state.

This procedure enables the removal of the relative time, $r^{0}$, in the bilocalized theory, essentially by canonical renormalization. The bilocal kinetic term contains a constraint that leads to a static internal field, $\phi \left(r\right)\to \phi \left(\vec{r}\right)$, in the center-of-mass frame. Hence, we obtain a static Schrödinger–Klein–Gordon (SKG) equation for the internal wave function. The eigenvalue of this equation is the $m^{2}$ of the bound state.

The SKG equation likewise contains a static potential that comes from the Feynman propagator of the exchanged particle in the parent theory. Typically we have a Yukawa potential, $∼g^{2}Me^{-2Mr}/8\pi r$, though the formalism can in principle accommodate any desired phenomenological potential. Here, *g* is the exchanged particle coupling constant, such as the coloron coupling (massive perturbative gluon) for fermions. This is not the scalar–fermion Yukawa coupling, $g_{Y}$, which is subsequently emergent.

We find that the Yukawa potential is classically critical with coupling $g_{c}$. If the coupling is sub-critical, $g<g_{c}$, then $m^{2}$ is positive, and the bound state is therefore a resonance. It will decay to its constituents if kinematically allowed. $\phi (\vec{r})$ is then a localized “lump”, with a radiative tail representing the two body decay and production by external free particles.

If the coupling is supercritical, $g>g_{c}$, then $m^{2}<0$ is tachyonic and Φ will acquire a VEV. We require an interaction, such as ${∼\lambda |\Phi |}^{4}$, to stabilize the vacuum and we therefore have spontaneous symmetry breaking. ϕ(r) is expected to be localized in its potential and χ(X) acquires the VEV. If ϕ(r) becomes delocalized, both χ(X) and ϕ(r) acquire VEVs, which is an analogue of a Bose–Einstein condensate, e.g., in a slightly heated superconductor; however, we have not produced solutions to the SKG equation that demonstrates this behavior.

We consider a bound state of chiral fermions in the coloron model, where a coloron is a massive gluon with coupling *g*, such as in “topcolor” models [11,12] and chiral constituent quark models [13]. Fierz’s rearrangement of the non-local, color-current-current, interaction yields a leading large $N_{c}$ interaction in $\bar{\psi }{\left(x\right)}_{L}\psi {\left(y\right)}_{R}\;∼\;\Phi \left(x,y\right)$. For the color singlet, Φ, the coupling $g^{2}$ is enhanced by $N_{c}$ in analogy to a BCS superconductor [19,20].

As our main result, we find that the coloron model can be classically critical. The critical coupling, $g_{c}^{2}$, extracted from [28,29], is astonishingly close to the critical value of the Yukawa coupling in the NJL model. While in the NJL model the critical behavior is O(ℏ) coming from fermion loops, in the bilocal model this is a semiclassical result, and the essential factor of $N_{c}$ comes from the coherent BCS-like enhancement of the four-fermion scattering amplitudes. It is therefore unclear what happens when we include the fermion loops in addition to the classical behavior in the large $N_{c}$ limit. Is criticality further enhanced by the additional loop contribution? Is there an additional $N_{c}$ enhancement of the underlying coupling due to the $\sqrt{N_{c}}$ factor in the emergent Yukawa interaction? These are interesting issues we will address elsewhere.

The induced Yukawa coupling of the bound state to fermions, $g_{Y}\bar{\psi }{\left(x\right)}_{L}\psi {\left(y\right)}_{R}\Phi \left(x,y\right)$, is extended and emergent in the composite models. We derive the coupling and find $g_{Y}\propto \int V\left(r\right)\phi \left(r\right)∼g^{2}\phi \left(0\right)$ in the point-like limit. For ϕ(0), in a tiny spherical cavity, we obtain $g_{Y}=g^{2}/g_{cNJL}$. Hence, the critical value of $g^{2}$ implies the critical value of $g_{Y}=g_{cNJL}$ in the point-like limit NJL model, which is consistent.

However, the ∫V(r)ϕ(r) could in principle be reduced to an extended potential as the wave function spreads out, even if $g^{2}$ is critical. This is an intriguing possibility: the BEH–Yukawa coupling of the top quark is $g_{top}∼1$, which is perturbative and is insufficient to drive the formation of a composite, negative $m^{2}$, Brout–Englert–Higgs (BEH) boson at low energies in, e.g., a top condensation model. However, the present result suggests that perhaps ∫V(r)ϕ(r) is small suppressing $g_{top}$, even though the underlying coloron coupling, $g^{2}N_{c}$, is super-critical and leads to the composite BEH mechanism. In this picture, the BEH boson may be a large object, e.g., a “balloon” of size $∼m_{top}^{-1}$ (see [31]).

While we have an eye to a composite BEH boson for the standard model, as in top condensation theories [22,23,24,32,33], our present analysis is more general, but does not yet include many details, e.g., gauge interactions and gravity. We think the emphasis on a bosonic field description, the treatment of the coloron model and its classical criticality, its linkage the NJL model as UV completion, and our treatment of relative time renormalization comprises a novel perspective.

## Data Availability

Data is contained within the article.

## Appendix A. Summary of Notation

Two body kinematics:

(A1) $$\begin{array}{ccc} & & p_{1}x+p_{2}y=\left(p_{1}+p_{2}\right)X+\left(p_{1}-p_{2}\right)r\\ & & p_{1}^{2}=p_{2}^{2}=\mu^{2}\;P=\left(p_{1}+p_{2}\right)\;Q=\left(p_{1}-p_{2}\right)\\ & & P^{2}+Q^{2}=4\mu^{2};\;\mathrm{rest}\;\mathrm{frame}:\;P^{0}=2p_{1}^{0};\;\vec{Q}=2{\vec{p}}_{1}; \end{array}$$

$P^{2}={\left(p_{1}+p_{2}\right)}^{2}$, though commonly referred to as the “invariant mass” of a pair, is not a scale breaking mass in that it involves fields with a traceless stress tensor.

Barycentric coordinates:

(A2) $$\begin{array}{ccc} & & X=\frac{1}{2}\left(x+y\right)\;\rho =\left(x-y\right)\;r=\frac{1}{2}\left(x-y\right)\\ & & \partial_{x}=\frac{1}{2}\left(\partial_{X}+\partial_{r}\right)=\frac{1}{2}\partial_{X}+\partial_{\rho }\\ & & \partial_{y}=\frac{1}{2}\left(\partial_{X}-\partial_{r}\right)=\frac{1}{2}\partial_{X}-\partial_{\rho } \end{array}$$

Two-body scattering states

(A3) $$\begin{array}{ccc} & & \Phi \left(x,y\right)=\exp\left(ip_{1}x+ip_{2}y\right)=\exp\left(iP_{\mu }X^{\mu }+iQ_{\mu }r^{\mu }\right)\\ & & \left(\partial_{x}^{2}+\partial_{y}^{2}\right)\Phi \left(x,y\right)=\left(p_{1}^{2}+p_{2}^{2}\right)\Phi \left(x,y\right)=2\mu^{2}\Phi \left(x,y\right)\\ & & =\frac{1}{2}\left(\frac{\partial^{2}}{\partial X^{\mu }\partial X_{\mu }}+\frac{\partial^{2}}{\partial r^{\mu }\partial r_{\mu }}\right)\Phi \left(X,r\right)=2\mu^{2}\Phi \left(x,y\right)\\ & & \to \left(\frac{\partial^{2}}{\partial X^{\mu }\partial X_{\mu }}+\frac{\partial^{2}}{\partial r^{\mu }\partial r_{\mu }}\right)\Phi \left(X,r\right)=4\mu^{2}\\ & & \left(\partial_{x}^{2}-\partial_{y}^{2}\right)\Phi \left(x,y\right)=\frac{\partial^{2}}{\partial X^{\mu }\partial r_{\mu }}\Phi \left(X,r\right)=0\\ & & dx^{2}+dy^{2}=2dX^{2}+\frac{1}{2}d\rho^{2}=2dX^{2}+2dr^{2}\\ & & \partial_{x}^{2}+\partial_{y}^{2}=\frac{1}{2}\partial_{X}^{2}+\frac{1}{2}\partial r^{2}=\frac{1}{2}\partial_{X}^{2}+2\partial_{\rho }^{2}\\ & & \partial_{x}^{2}-\partial_{y}^{2}=\partial_{X}^{\mu }\partial_{\mu r}=2\partial_{X}^{\mu }\partial_{\mu \rho } \end{array}$$

Integration measures

(A4) $$\begin{array}{ccc} & & \int_{u\ldots v}\;\;\;\;=\int \;\;d^{4}u\ldots d^{4}v\;\;\int_{\vec{x}\ldots \vec{y}}\;\;\;\;=\int \;\;d^{3}x\ldots d^{3}y\\ & & \int_{u\ldots v;\vec{x}\ldots \vec{y}}\;\;\;\;=\int \;\;d^{4}u..d^{4}v\;d^{3}x\ldots d^{3}y\\ & & \int_{u\ldots v;\vec{x}\ldots \vec{y}}^{\prime }\;\;\;\;=\int \;\;M^{4}d^{4}u..M^{4}d^{4}v\;M^{3}d^{3}x\ldots M^{3}d^{3}y\\ & & \int d^{4}xd^{4}y=\;\;\int d^{4}Xd^{4}\rho =J\;\;\int d^{4}Xd^{4}r;\\ & & \mathrm{Jacobian}\;\;J={\left(2\right)}^{4}\\ & & \int d^{4}xd^{4}y\left(|\partial_{x}{\phi |}^{2}+|\partial_{y}{\phi |}^{2}-\mu^{2}{\left|\phi \right|}^{2}\right)\\ & & =J\;\;\int d^{4}Xd^{4}r\left(\frac{1}{2}|\partial_{X}{\phi |}^{2}+\frac{1}{2}|\partial_{r}{\phi |}^{2}-\mu^{2}{\left|\phi \right|}^{2}\right)\\ & & =\int d^{4}Xd^{4}\rho \left(\frac{1}{2}|\partial_{X}{\phi |}^{2}+2|\partial_{\rho }{\phi |}^{2}-\mu^{2}{\left|\phi \right|}^{2}\right)\\ & & \mathrm{Hermitian}\;\mathrm{operator}:\;\;W+W^{†}=W_{+h.c.} \end{array}$$

## Appendix B. Bilocal Field Theory

Here, we give a general discussion of our “revisited” bilocal field theory for a pair of particles, as inspired by Yukawa [1,2,3]. We begin with the “bilocalization” and subsequently construct the generic actions for bilocal fields containing free particles.

### Appendix B.1. Free Fields

Let us examine the bilocalization procedure in Section for free particle states. Consider a pair of local scalar fields $\varphi_{i}\left(x\right)$ (complex):

(A5) $$\begin{array}{ccc} & & \;\;\;\;\;\;\;\;\;\;\;\;\;\;\;\;\;\;\;\;S=\int_{x}(|\partial \varphi_{1}{|}^{2}+|\partial \varphi_{2}{|}^{2}-\mu^{2}\left(\right|\varphi_{1}{|}^{2}+|\varphi_{2}{|}^{2}) \end{array}$$

with independent free particle equations of motion

(A6) $$\begin{array}{ccc} & & \partial^{2}\varphi_{1}+\mu^{2}\varphi_{1}=0\;\partial^{2}\varphi_{2}+\mu^{2}\varphi_{2}=0 \end{array}$$

We want to describe a pair of particles by a bilocal field of mass dimesion 1:

(A7) $$\begin{array}{ccc} & & \Phi \left(x,y\right)=M^{-1}\varphi_{1}\left(x\right)\varphi_{2}\left(y\right) \end{array}$$

We therefore have the two equations of motion for Φ

(A8) $$\begin{array}{ccc} & & \left(\partial_{x}^{2}+\partial_{y}^{2}\right)\Phi \left(x,y\right)+2\mu^{2}\Phi \left(x,y\right)=0 \end{array}$$

(A9) $$\begin{array}{ccc} & & \left(\partial_{x}^{2}-\partial_{y}^{2}\right)\Phi \left(x,y\right)=0. \end{array}$$

### Appendix B.2. Bilocalization of Scattering States

We can obtain the action for Φ as follows. We multiply the kinetic and mass terms of Equation (A5) by world scalars:

(A10) $$\begin{array}{ccc} & & W_{i}=ZM^{2}\int \;\;d^{4}y\;{\left|\varphi_{i}\left(y\right)\right|}^{2} \end{array}$$

and hence,

(A11) $$\begin{array}{ccc} & & \;\;\;\;\;\;\;\;\;\;S=\int_{x}\left(W_{2}|\partial \varphi_{1}{|}^{2}+W_{1}|\partial \varphi_{2}{|}^{2}-\mu^{2}(W_{2}|\varphi_{1}{|}^{2}+W_{1}|\varphi_{2}{|}^{2})\right)\\ & & \;\;\;\;\;\;\;\;\;\;=ZM^{2}\;\;\int_{xy}\;\;(|\varphi_{2}\left(y\right)\partial_{x}\varphi_{1}{\left(x\right)|}^{2}+{\left|\varphi_{1}\left(y\right)\partial_{x}\varphi_{2}\left(x\right)\right|}^{2}\\ & & \;\;-2\mu^{2}{\left|\varphi_{1}\left(y\right)\varphi_{2}\left(x\right)\right|}^{2}) \end{array}$$

At this stage, we can still vary with respect to either $\varphi_{1}$ of $\varphi_{2}$, and the equations are modified. In terms of the bilocal field, we have

(A12) $$\begin{array}{ccc} & & \;\;\;\;\;\;\;\;\;\;=ZM^{4}\;\;\int_{xy}\;\;\left(|\partial_{x}{\Phi \left(x,y\right)|}^{2}+|\partial_{y}{\Phi \left(x,y\right)|}^{2}-2\mu^{2}{\left|\Phi \left(x,y\right)\right|}^{2}\right) \end{array}$$

We go to barycentric coordinates and note the derivatives:

(A13) $$\begin{array}{ccc} & & X=(x+y)/2\;\;r=(x-y)/2\\ & & \partial_{x}=\left(\partial_{X}+\partial_{r}\right)/2\;\partial_{y}=\left(\partial_{X}-\partial_{r}\right)/2\\ & & \Phi (x,y)\to \Phi (X,r) \end{array}$$

The factor of *M* is superfluous at this point, and in what follows we can set M=1 (we’ll restore it below). Hence:

(A14) $$\begin{array}{ccc} & & \;\;\;\;\;\;\;\;\;\;\;\;S=\;JZ\;\;\int_{Xr}\;\;(\;\frac{1}{4}|\left(\partial_{X}+\partial_{r}\right)\Phi \left(X,r\right){|}^{2}+\frac{1}{4}{\left|\left(\partial_{X}-\partial_{r}\right)\Phi \left(X,r\right)\right|}^{2}\\ & & -2\mu^{2}{\left|\Phi \left(X,r\right)\right|}^{2}) \end{array}$$

which yields the action

(A15) $$\begin{array}{ccc} & & \;\;\;\;\;\;\;\;\;\;\;S=\;\frac{JZ}{2}\;\;\int_{Xr}\;\;\left(|\partial_{X}{\Phi \left(X,r\right)|}^{2}+|\partial_{r}{\Phi \left(X,r\right)|}^{2}-4\mu^{2}{\left|\Phi \left(X,r\right)\right|}^{2}\right) \end{array}$$

If we vary $\delta \Phi \left(x^{\prime },y^{\prime }\right)=\delta^{4}\left(x-x^{\prime }\right)\delta^{4}\left(y-y^{\prime }\right)$, we obtain one equation of motion:

(A16) $$\begin{array}{ccc} & & \partial_{X}^{2}\Phi \left(X,r\right)+\partial_{r}^{2}\Phi \left(X,r\right)+4\mu^{2}\Phi \left(X,r\right)=0 \end{array}$$

We see that this is consistent with Equation (A8)

(A17) $$\begin{array}{ccc} & & \partial_{x}^{2}+\partial_{y}^{2}=\frac{1}{2}\left(\partial_{X}^{2}+\partial_{r}^{2}\right). \end{array}$$

However, Equation (A9) is missing. We see using

(A18) $$\begin{array}{ccc} & & \left(\partial_{x}^{2}-\partial_{y}^{2}\right)=\frac{\partial }{\partial X^{\mu }}\frac{\partial }{\partial r^{\mu }} \end{array}$$

that Equation (A9) takes the form

(A19) $$\begin{array}{ccc} & & \frac{\partial }{\partial X^{\mu }}\frac{\partial }{\partial r^{\mu }}\Phi \left(X,r\right)=0 \end{array}$$

As before, this can be viewed as a constraint, and we can treat it as a Lagrange multiplier as in Equation (28) to supply the second equation.

We factorize Φ in barycentric coordinates as

(A20) $$\begin{array}{ccc} & & \sqrt{J/2}\;\Phi \left(X,r\right)=\chi \left(X\right)\phi \left(r\right) \end{array}$$

The action Equation (A15) becomes

(A21) $$\begin{array}{ccc} & & \;\;\;\;\;\;\;\;\;\;\;\;\;\;\;\;\;\;\;\;S=Z\;\;\int_{Xr}{\;\;(|\phi \left(r\right)|}^{2}{\left|\partial_{X}\chi \left(X\right)\right|}^{2}\\ & & \;+{\left|\chi \left(X\right)\right|}^{2}\left(\right|\partial_{r}{\phi \left(r\right)|}^{2}-4\mu^{2}{\left|\phi \left(r\right)\right|}^{2})) \end{array}$$

We then define

(A22) $$\begin{array}{ccc} & & 1=Z\int d^{4}r{\left|\phi \left(r\right)\right|}^{2} \end{array}$$

The Schrödinger–Klein–Gordon equation has eigenvalue $m^{2}$

(A23) $$\begin{array}{ccc} & & \;\;\;\;\;\;\;\;\;\;\partial_{r}^{2}\phi \left(r\right)+4\mu^{2}\phi \left(r\right)=m^{2}\phi \left(r\right)\\ & & \;\;\;\;\;\;\;\;\;\;m^{2}\int_{r}{\left|\phi \left(r\right)\right|}^{2}=\int_{r}\left(\right|\partial_{r}{\phi \left(r\right)|}^{2}+4\mu^{2}{\left|\phi \left(r\right)\right|}^{2}) \end{array}$$

Then, the χ equation becomes

(A24) $$\begin{array}{ccc} & & \partial_{X}^{2}\chi \left(X\right)+m^{2}\chi \left(X\right)=0 \end{array}$$

The constraint then takes the form

(A25) $$\begin{array}{ccc} & & \frac{\partial \chi (X)}{\partial X^{\mu }}\frac{\partial \phi (r)}{\partial r^{\mu }}=0 \end{array}$$

In the barycentric (rest) frame, we can choose χ to have four-momentum $P_{\mu }=\left(m,0,0,0\right)$. The constraint becomes $P_{\mu }\partial_{r}^{\mu }\phi \left(r\right)=0$, Therefore, ϕ becomes a *static function* of $r^{\mu }=\left(0,\vec{r}\right)$. The Schrödinger–Klein–Gordon equation is then a static equation

(A26) $$\begin{array}{ccc} & & -\nabla_{\vec{r}}^{2}\phi \left(\vec{r}\right)+4\mu^{2}\phi \left(\vec{r}\right)=m^{2}\chi \left(\vec{r}\right) \end{array}$$

We define the static internal wave function $\phi (\vec{r})$ to be dimensionless, d=0, and we normalize the dimensionless static wave function (restoring *M*)

(A27) $$\begin{array}{ccc} & & \left(M^{3}\right)\int d^{3}\vec{r}\;{\left|\phi \left(\vec{r}\right)\right|}^{2}=1 \end{array}$$

We then have

(A28) $$\begin{array}{ccc} & & \;\;\;\;\;\;\;\;\;\;1=Z\left(M^{4}\right)\;\;\int_{r}|\phi \left(r\right){|}^{2}=Z\left(M^{4}\right)\;\;\int dr^{0}\int d^{3}\vec{r}{\left|\phi \left(\vec{r}\right)\right|}^{2}=ZT\left(M\right) \end{array}$$

where an integral over relative time has appeared leading to an overall factor ZT(M)=1 in the action for χ. The action becomes two nested parts

(A29) $$\begin{array}{ccc} & & \;\;\;\;\;\;\;\;\;\;S=\;\;\int_{X}\;\;\left(|\partial_{X}{\chi \left(X\right)|}^{2}-m^{2}{\left|\chi \left(X\right)\right|}^{2}\right)\\ & & \;\;\;\;\;\;\;\;\;\;m^{2}\;\;\int_{\vec{r}}\;\;d^{3}\vec{r}\;|\phi \left(\vec{r}\right){|}^{2}=\int_{\vec{r}}\left(\right|\nabla_{\vec{r}}\phi \left(r\right){|}^{2}+4\mu^{2}|\phi \left(r\right){|}^{2}) \end{array}$$

with Equation (A27).

### Appendix B.3. Kinematics of Scattering States

We can more directly construct the bilocal fields, without appealing to world scalars, by considering simple free particle kinematics. For free particles of four-momenta $p_{i}$, we see that Φ describes a scattering state

(A30) $$\begin{array}{ccc} & & \Phi \left(x,y\right)∼\exp\left(ip_{1\mu }x^{\mu }+ip_{2\mu }y^{\mu }\right)=\exp\left(iP_{\mu }X^{\mu }+iQ_{\mu }r^{\mu }\right) \end{array}$$

where

(A31) $$\begin{array}{ccc} & & P_{\mu }=p_{1\mu }+p_{2\mu }\;Q_{\mu }=p_{1\mu }-p_{2\mu } \end{array}$$

For massive particles, $p_{1}^{2}=p_{2}^{2}=\mu^{2}$, Φ satisfies

(A32) $$\begin{array}{ccc} & & \;\;\;\;\;\;\;\;\;\;\;\;\;\;\;\;\;\;\;\;\;\;\;\;\left(\frac{\partial^{2}}{\partial X^{\mu }\partial X_{\mu }}\;\;+\;\;\frac{\partial^{2}}{\partial r^{\mu }\partial r_{\mu }}\;\;+\;4\mu^{2}\right)\Phi \left(X,r\right)=0 \end{array}$$

The constraint is the second equation

(A33) $$\begin{array}{ccc} & & \;\;\;\;\;\;\;\;\;\;\;\;\;\;\;\;\;\;\;\;\;\;\;\;\left(\partial_{x}^{2}-\partial_{y}^{2}\right)\Phi \left(x,y\right)=\frac{\partial^{2}}{\partial X^{\mu }\partial r_{\mu }}\Phi \left(X,r\right)=0 \end{array}$$

and forces $\Phi (X,\vec{r})$ to have $r^{0}=0$ in the center-of-mass frame; hence, $P_{\mu }Q^{\mu }=0$ and $Q=(0,\vec{q})$.

**Constant Positive $m_{0}^{2}=4\mu^{2}$**:

In the center-of-mass frame, $p_{1}^{0}=p_{2}^{0}$, $Q^{0}=0$,

(A34) $$\begin{array}{ccc} & & Q^{2}={\left(p_{1}-p_{2}\right)}^{2}=-{\left({\vec{p}}_{1}-{\vec{p}}_{2}\right)}^{2}=-{\vec{q}}^{\;2} \end{array}$$

Hence, from Equations (A32) and (A33)

(A35) $$\begin{array}{ccc} & & P^{2}=4\mu^{2}+{\vec{q}}^{\;2}\\ & & \frac{\partial^{2}}{\partial X^{\mu }\partial X_{\mu }}\Phi \left(X,\vec{r}\right)=\left(m_{0}^{2}+{\vec{q}}^{\;2}\right)\Phi \left(X,\vec{r}\right) \end{array}$$

Therefore, if we were to try to interpret Φ as a “bound state”, we see that is has continuum of invariant “masses” $m^{2}=m_{0}^{2}+{\vec{q}}^{\;2}$. This is evidently an “unparticle” [34]. This implies it is not localized and is a scattering state of asymptotic free particles each of mass $\mu =m_{0}/2$.

With the constraint of Equation (A33), we can choose $r^{\mu }=\left(0,\vec{r}\right)$ and $X^{\mu }=\left(X^{0},0\right)$. We factorize Φ=χ(X)ϕ(r) and the factor field ϕ(r) then satisfies the static SKG equation with eigenvalue $M^{2}$

(A36) $$\begin{array}{ccc} & & -\nabla_{\vec{r}}^{2}\phi \left(\vec{r}\right)+m_{0}^{2}\phi \left(\vec{r}\right)=m^{2}\phi \left(\vec{r}\right) \end{array}$$

the solutions of the factorized field ϕ(r) are static, box normalized, plane waves, with eigenvalues $m^{2}\equiv m_{\vec{q}}^{2}=m_{0}^{2}+{\vec{q}}^{\;2}$

(A37) $$\begin{array}{ccc} & & \phi \left(\vec{r}\right)=\frac{1}{\sqrt{V}}\exp\left(i\vec{q}\cdot \vec{r}\right)\;m_{\vec{q}}^{2}={\vec{q}}^{\;2}+m_{0}^{2} \end{array}$$

χ(X) then satisfies the KG equation with $X^{0}=t$ and $\vec{X}=0$

(A38) $$\begin{array}{ccc} & & \partial_{t}^{2}\chi \left(t\right)+m_{\vec{q}}^{2}\chi \left(t\right)=0\;t=X^{0} \end{array}$$

and the χ(X) solution becomes

(A39) $$\begin{array}{ccc} & & \chi \left(X\right)\propto \exp\left(im_{\vec{q}}t\right) \end{array}$$

This is just the spectrum of the two body states of μ-massive particles in the barycentric frame.

**Constant Negative $m_{0}^{2}$**:

If we suppose $m_{0}^{2}<0$ then the solutions for Φ(t,r) for small $\vec{q}$ are runaway exponentials, $\Phi ∼\exp(|m_{0}|t)$. The eigenvalues are

(A40) $$\begin{array}{ccc} & & \;m_{\vec{q}}^{2}={\vec{q}}^{\;2}-{\left|m_{0}\right|}^{2} \end{array}$$

This analogous to a “Dirac sea” of negative mass eigenvalues extending from ${\vec{q}}^{\;2}=0$ to ${\vec{q}}^{\;2}={\left|m_{0}\right|}^{2}$, where the lowest mass state has $\vec{q}=0$ and eigenvalue $-|m_{0}{|}^{2}$.

This would therefore be a tachyonic instability of unparticles, and we then require higher field theoretic interactions, such as ${\lambda |\Phi |}^{4}$, to stabilize the vacuum. This represents a spontaneous breaking of the chiral symmetry, and the constituent fermions will dynamically acquire masses and the instability is halted.

However, a constant negative $m_{0}^{2}$ does not give a Higgs-like spontaneous symmetry breaking mechanism. If the potential is

(A41) $$\begin{array}{ccc} & & V=-m_{0}^{2}{\left|\Phi \right|}^{2}+\frac{\lambda }{2}{\left|\Phi \right|}^{4} \end{array}$$

where we have constant $-m_{0}^{2}$ rather than a localized potential. Then, indeed, the field develops a constant VEV:

(A42) $$\begin{array}{ccc} & & \langle \Phi \rangle =V+\Phi^{0}\;m_{0}^{2}=v^{2}\lambda \end{array}$$

Then, $\Phi^{0}$ indeed becomes a massive BEH mode, but with the continuous spectrum $m^{2}={\left|m_{0}\right|}^{2}+{\vec{q}}^{\;2}$. To have an acceptable BEH mechanism, with a well-defined BEH boson, we therefore require a localized potential in $\vec{r}$ where $\langle \Phi \rangle =\langle \chi \rangle \phi \left(\vec{r}\right)$ and $\phi (\vec{r})$ is a localized eigenmode.

### Appendix B.4. Removal of Relative Time and Generic Potential

A point-like interaction in the underlying theory may produce an effective action with a potential term that is a function of $r^{\mu }$

(A43) $$\begin{array}{ccc} & & \;\;\;\;\;\;\;\;\;\;\;\;\;\;\;\;\;\;\;\;S=\frac{JM^{4}}{2}\;\;\int_{Xr}\;\;\;(Z|\partial_{X}{\Phi |}^{2}+Z|\partial_{r}{\Phi |}^{2}\;-U\left(2r^{\mu }\right)|\Phi \left(X,r\right){|}^{2}) \end{array}$$

and we assume the constraint:

(A44) $$\begin{array}{ccc} & & \;\;\;\;\;\;\;\;\;\;\;\;\;\;\;\;\;\;\;\;S=\;\;\int_{Xr}\eta {\left(\frac{\partial \Phi^{†}}{\partial X^{\mu }}\frac{\partial \Phi }{\partial r_{\mu }}+h.c.\right)}^{2}. \end{array}$$

We factorize Φ

(A45) $$\begin{array}{ccc} & & \sqrt{J/2}\;\Phi \left(X,r\right)=\chi \left(X\right)\phi \left(r\right) \end{array}$$

In the center-of-mass frame we impose the constraint, $\Phi \left(X,r\right)\to \chi \left(X^{0}\right)\phi \left(\vec{r}\right)$. We can then integrate over relative time

(A46) $$\begin{array}{ccc} & & \;\;\;\;\;\;\;\;\;\;\;\;\;\;\;\;\;\;\;\;S=M^{3}\;\;\int_{X\vec{r}}\left(ZTM\right|\partial_{X}{\chi |}^{2}|\phi \left(\vec{r}\right){|}^{2}-ZTM|\chi \left(X\right){|}^{2}{\left|\nabla_{\vec{r}}\phi \right|}^{2}\\ & & -V\left(\vec{r}\right)|\chi \left(X\right){|}^{2}{\left|\phi \left(\vec{r}\right)\right|}^{2}) \end{array}$$

where

(A47) $$\begin{array}{ccc} & & V\left(\vec{r}\right)=M\int dr^{0}U\left(2r^{\mu }\right) \end{array}$$

and $V(\vec{r})$ has dimensions of $M^{2}$. We now define the normalizations,

(A48) $$\begin{array}{ccc} & & ZTM=1\;\;\;\;1=M^{3}\;\;\int \;\;d^{3}r{\left|\phi \left(\vec{r}\right)\right|}^{2}. \end{array}$$

The purpose of these normalizations is to have a canonically normalized χ field in the center-of-mass frame, even in the limit g=0. It also defines the conserved χ current to have unit charge (one boundstate pair) to match the underlying theory’s conserved current.

Hence, we obtain the actions:

(A49) $$\begin{array}{ccc} & & \;\;\;\;\;\;\;\;\;\;S=\;\;\int_{X}\;\;\left(|\partial_{X}\chi \left(X\right)|-m^{2}{\left|\chi \left(X\right)\right|}^{2}\right)\\ & & \;\;\;\;\;\;\;\;\;\;m^{2}\;\;\int_{\vec{r}}\;\;d^{3}\vec{r}\;|\phi \left(\vec{r}\right){|}^{2}=\int_{\vec{r}}\left(\right|\nabla_{\vec{r}}\phi \left(r\right){|}^{2}+V\left(r\right)|\phi \left(r\right){|}^{2}) \end{array}$$

with Equation (A48).

An attractive (repulsive) potential is V<0 (V>0). The main point is that the field χ(X) must have canonical normalization of its kinetic term, which dictates the introduction of *Z*. The kinetic term of χ is seen to be extensive in *T*, and this is absorbed by normalizing *Z*, as ZMT=1. The potential is determined by the integral over relative time of the interaction and is not extensive in *T*.

From the factorization $\Phi =\chi \left(X\right)\phi \left(\vec{r}\right)$ with normalized ϕ(r), as in Equation (38), the eigenvalue $m^{2}$ is computed in the rest frame

(A50) $$\begin{array}{ccc} & & \;\;\;\;\;\;\;\;\;\;\;\;\;\;\;\;\;\;\;\;m^{2}=m^{2}\int_{\vec{r}}{\left|\phi \left(\vec{r}\right)\right|}^{2}=\;\;\int_{\vec{r}}\left(-\phi^{†}\nabla^{2}\phi +V\left(\vec{r}\right){\left|\phi \left(\vec{r}\right)\right|}^{2}\right) \end{array}$$

From this, we obtain a normalized effective action for χ in any frame

(A51) $$\begin{array}{ccc} & & S_{\chi }=\int d^{4}X\left(\frac{\partial \chi^{†}}{\partial X^{\mu }}\frac{\partial \chi }{\partial X_{\mu }}-m^{2}{\left|\chi \right|}^{2}\right) \end{array}$$

The mass of the bound state is determined by the eigenvalue of the static Schrödinger–Klein–Gordan (SKG) equation in the center-of-mass frame:

(A52) $$\begin{array}{ccc} & & -\nabla^{2}\phi +V\left(\vec{r}\right)\phi \left(\vec{r}\right)=m^{2}\phi \left(\vec{r}\right). \end{array}$$
